# Respuesta clínica a brivaracetam en dos casos de epilepsia de ausencia juvenil farmacorresistentes

**DOI:** 10.23938/ASSN.1132

**Published:** 2025-09-22

**Authors:** Abel Alejandro Sanabria Sanchinel, Byron Daniel Bol Marroquín

**Affiliations:** Humana: Centro de Epilepsia y Neurocirugía funcional Ciudad de Guatemala Guatemala

**Keywords:** Epilepsia, Epilepsia de Ausencia Juvenil, Epilepsia Refractaria, Valproato Sódico, Brivaracetam, Epilepsy, Epilepsy, Absence, Drug Resistant Epilepsy, Valproic Acid, Brivaracetam

## Abstract

Si bien solo el 20% de las personas con epilepsias generalizadas idiopáticas son farmacorresistentes, dicho porcentaje resulta bastante problemático si se toma en cuenta los escasos fármacos aprobados para este tipo de epilepsia. Solo el ácido valproico y la etosuximida tienen aprobación por la *Food and Drug Administration* y la *European Medicines Agency* para el tratamiento de las crisis de ausencia, además de la lamotrigina por la *European Medicines Agency.* Existe bajo grado de evidencia para el uso del levetiracetam en este tipo de crisis, y el brivaracetam aún no tiene ninguna aprobación para su uso en epilepsias generalizadas.

Presentamos los casos de dos mujeres con epilepsia de ausencia juvenil con respuesta incompleta e intolerancia a tratamientos de primera línea, que experimentaron una mejoría importante con el tratamiento con brivaracetam.

## Abstract (en)

Although only around 20% of individuals with idiopathic generalized epilepsy are drug-resistant, this minority presents a significant therapeutic challenge due to the limited number of approved medications for this condition. For absence seizures, only valproic acid and ethosuximide are approved by both the U.S. Food and Drug administration and the European Medicines Agency, with lamotrigine additionally approved by the European Medicines Agency. Evidence supporting the use of levetiracetam in this seizure type is limited, and brivaracetam has not yet been approved for generalized epilepsy.

We report two cases of juvenile absence epilepsy in women with incomplete response and intolerance to first-line treatments, who demonstrated marked clinical improvement following treatment with brivaracetam.

## INTRODUCCIÓN

Las epilepsias generalizadas idiopáticas incluyen la epilepsia de ausencia juvenil, la epilepsia de ausencias de la niñez, la epilepsia mioclónica juvenil, y la epilepsia generalizada genética con solo crisis generalizadas tónico-clónicas^1^. Los fármacos aprobados para tratar las crisis presentes en este tipo de epilepsia son escasos: ácido valproico (VPA), lamotrigina (LTG), etosuximida (ESM), topiramato (TPM), levetiracetam (LEV), perampanel (PER) y benzodiacepinas^2^; un 20% de las personas con epilepsias generalizadas idiopáticas presenta farmacorresistencia^3^.

Las personas con epilepsia de ausencia juvenil disponen de un arsenal aún más restringido para tratar sus crisis de ausencia: VPA y ESM, aprobados tanto por la *Food and Drug Administration* (FDA) como por la *European Medicines Agency* (EMA), y LTG aprobado solamente por la EMA^2^.

El brivaracetam (BRV) es una pirrolidona derivada estructuralmente del LEV y del piracetam, una de cuyas características diferenciales es su mayor afinidad por la proteína SV2A^4^, hasta 30 veces más que el LEV. La proteína neuronal específica SV2A es una proteína transmembrana muy abundante en las vesículas sinápticas que regula su fusión con la membrana presináptica y la liberación de los neurotransmisores al espacio sináptico^4^. Está aprobado por la FDA y por la EMA como tratamiento para epilepsias de inicio focal en personas desde los cuatro años de edad^4^. Sin embargo, los datos experimentales previos a su aprobación y su extendido uso posterior a su comercialización han demostrado suficiente eficacia para el tratamiento de las epilepsias generalizadas idiopáticas^5^^-^^9^.

Dada la posibilidad de encontrar pacientes con epilepsias generalizadas fármaco resistentes, es importante considerar otros fármacos que puedan tener respuesta en este tipo de epilepsia. Es por ello que a continuación presentamos dos casos de epilepsia de ausencia juvenil con respuesta a BRV.

## CASOS CLÍNICOS

### Caso 1

Mujer de 16 años, diestra, sin otros antecedentes de interés. Desde los 13 años presentaba eventos de alteración de la consciencia que no se precedían ni acompañaban de otras manifestaciones, con una recuperación completa posterior; eran eventos breves, de 15 a 30 segundos de duración, con frecuencia de dos a tres por día, compatibles con crisis de ausencia (CA). También presentaba crisis tónico clónicas generalizadas (CTCG) de dos a tres minutos de duración, con frecuencia de una vez por semana.

Se realizó una resonancia magnética cerebral que fue normal, y un electroencefalograma (montaje longitudinal bipolar) de una hora que evidenció actividad epiléptica interictal generalizada en forma de punta-onda lenta a Hz, durante la lectura en lengua extranjera (material suplementario). Se inició LEV con pauta ascendente hasta 1.000 mg cada 12 horas, e inicialmente se obtuvo control total de las CTCG y las CA se redujeron a una por semana. Luego de un año con buen control de las crisis, continuó sin CTCG pero las CA aumentaron a ocho crisis por día, tres a cuatro días por semana, y se notaron dificultades académicas. Por ser mujer en edad fértil, se planteó asociar LTG y se produjo una reacción alérgica con manifestaciones cutáneas durante el protocolo de incremento de dosis, por lo que debió retirarse.

Luego se inició tratamiento con VPA de liberación inmediata, 500 mg cada 12 horas, dosis que no toleró debido a que le provocó malestar general por lo que bajó por cuenta propia a 500 mg cada 24 horas. La dosis tolerada de VPA permitió que permaneciera sin crisis de ausencia durante seis meses.

Tras ese período se reiniciaron las CTCG, con frecuencia de una a cuatro en un mes y las CA, que aparecían tres a cuatro días por semana. Los niveles plasmáticos de VPA eran de 94,1 μg/mL, y los niveles de amonio de 86 μg/dL.

Se intentó aumentar la dosis de LEV, pero le produjo irritabilidad.

Se le planteó realizar *switch* de LEV a BRV, y aceptó, se realizó con relación 10:1. Tras la realización del *switch* presentó solamente una CTCG cuatro días después del cambio, y luego con frecuencia de una vez cada dos meses, y períodos de semanas completas sin presentar CA. Permaneció en este estado durante un período de 6 meses hasta que, por motivos económicos, precisó cambiar nuevamente de BRV a LEV. Tras este cambio se incrementó la frecuencia de las CTCG a dos por mes, y las CA se presentaron con frecuencia al menos semanal. Se inició dieta cetogénica, con lo que se obtuvo nuevamente reducción de las CTCG y CA, a una crisis cada dos meses y ninguna crisis, respectivamente, desde el inicio de la dieta.

### Caso 2

Mujer de 28 años, diestra, sin otros antecedentes de interés. Desde los 19 años presentaba eventos breves, de 30 a 60 segundos de duración, de alteración de la consciencia no precedidos de aura ni acompañados de automatismos orales o manuales ni de otra manifestación motora clara, y que al finalizar no se asociaban a confusión apreciable, con una recuperación completa prácticamente inmediata. Se tipificó el cuadro como CA y se prescribió VPA, con buena respuesta. Permaneció sin crisis durante tres años.

Tras ese período le plantearon cambiar de VPA a LEV. Tras realizar el cambio a LEV, reaparecieron las CA con frecuencia de tres por día y aparecieron CTCG de novo con frecuencia de una por mes. Por razones que se desconocen, le asociaron fenitoína (PHT) al LEV y, sorprendentemente, no existió una exacerbación de las crisis. El fármaco se retiró debido a que presentó un exantema maculopapular tras iniciar el tratamiento con PHT.

Tras ser evaluada por primera vez en nuestro centro, se realizó un electroencefalograma (montaje referencial A1-A2) que evidenció actividad epiléptica interictal en forma de punta-onda lenta a 3.5 Hz de distribución generalizada con predominio anterior (material suplementario) y se le ofreció regresar a VPA. La paciente estaba en tratamiento con VPA 500 mg cada 12 horas y LEV 1.000 mg cada 12 horas, con niveles plasmáticos de VPA en 136 μg/mL, sin asociar efectos adversos, ni hiperamonemia, ni alteraciones de la función hepática. La paciente ya no presentó CTCG pero continuó con CA con frecuencia de diez crisis por semana.

Se retiró LEV por ineficacia, y permaneció con la misma frecuencia de crisis. Se le planteó iniciar tratamiento con LTG o TPM, pero la paciente rechazó estas opciones por los potenciales efectos adversos que se le comentaron. Se le planteó asociar BRV en dosis de 50 mg cada 12 horas, y aceptó. Tras comenzar con esta asociación de BRV y VPA, continuó sin CTCG y las CA se redujeron a una crisis por mes. En los últimos tres meses ha mantenido la misma pauta y mismo control de las crisis epilépticas.

## DISCUSIÓN

El BRV es un fármaco que ha demostrado una buena eficacia en los casos de epilepsia farmacorresistente, incluyendo una mejoría de la respuesta farmacológica en las epilepsias generalizadas idiopáticas y en pacientes que previamente no habían respondido a LEV^6^^,^^10^^-^^12^. Sin embargo, las muestras de pacientes con epilepsias generalizadas idiopáticas en estos estudios han sido consistentemente pequeñas. Se tiene la descripción de una respuesta excepcionalmente buena en dos pacientes con epilepsia con CA que habían sido refractarios a todos los fármacos para epilepsias generalizadas, aunque parte de los problemas con su uso habían sido la falta de adherencia y el uso de dosis inadecuadas dados sus efectos adversos^13^^,^^14^. Es importante tener en cuenta las limitaciones del BRV, y que el VPA es el fármaco de mayor eficacia en el control de las CA, seguido de ESM y LTG^6^. También es importante señalar que en el mercado de nuestro país, Guatemala, no está disponible la ESM, por lo que no es una opción terapéutica para nuestros pacientes, contexto que también puede darse en otros países. Entre otros factores a tener en cuenta con BRV, es que existe cierta evidencia sobre una menor incidencia de efectos adversos conductuales en comparación con LEV^4^^,^^14^.

En cuanto a sus interacciones farmacológicas, se ha documentado que fenobarbital, PHT y carbamazepina tienden a disminuir la concentración sérica de BRV, pero a niveles que no afectan al control de las crisis y que no requieren la modificación de las dosis^4^^,^^15^.

Dada la similitud del BRV con el LEV, y su mayor afinidad por la proteína SV2A, es válido considerar al BRV como un fármaco seguro de forma *off label* para el tratamiento de epilepsias generalizadas idiopáticas que no presentaron respuesta a los fármacos habituales, y con un perfil aceptable de efectos secundarios, como ha resultado en los dos casos presentados.

La epilepsia de ausencia juvenil es un tipo de epilepsia generalizada idiopática en la que contamos con pocas opciones terapéuticas con eficacia probada. El BRV, que en la actualidad solo tiene aprobación para epilepsia con crisis de inicio focal, es un fármaco que se puede considerar en aquellos casos de epilepsias generalizadas idiopáticas refractarios a los fármacos estándar, en casos donde no se tenga otra alternativa, siempre considerando esta aplicación *off label* y con un razonado juicio clínico. Se plantea la necesidad de que este fármaco sea estudiado en ensayos clínicos aleatorizados con una buena metodología y población suficiente para poder aprobar su uso en epilepsias generalizadas.

## Data Availability

Se encuentran disponibles bajo petición al autor de correspondencia.
